# The Emotional Divergent–Convergent Thinking Program (EDICOP): Design, Implementation, and Results

**DOI:** 10.3390/ejihpe10040074

**Published:** 2020-11-13

**Authors:** Goretti Soroa, Aitor Aritzeta, Alexander Muela, Nekane Balluerka, Arantxa Gorostiaga, Jone Aliri

**Affiliations:** 1 Department of Clinical and Health Psychology and Research Methodology, University of the Basque Country (UPV/EHU), 20018 San Sebastián, Gipuzkoa, Spain; alexander.muela@ehu.eus (A.M.); nekane.balluerka@ehu.eus (N.B.); arantxa.gorostiaga@ehu.eus (A.G.); jone.aliri@ehu.eus (J.A.); 2 Department of Basic Psychological Processes and Development, University of the Basque Country (UPV/EHU), 20018 San Sebastián, Gipuzkoa, Spain; aitor.aritzeta@ehu.eus

**Keywords:** creativity training, emotional education, program, higher education, assessment

## Abstract

In a social environment that requires young people to adapt to increasingly demanding situations, emotional education and creativity training may be key for both personal development and academic performance. Given that there are currently no known interventions that develop emotional and creative skills simultaneously in a youth population, the main objective of this study was to design, implement, and evaluate the Emotional Divergent–Convergent Thinking Program (EDICOP). The study design was quasi-experimental with a non-equivalent control group and pretest–posttest measures. The participants included 196 students between 16 and 24 years of age belonging to two centers of higher education. Our results showed that the EDICOP contributed to the improvement of the participants’ divergent-proactive style, positive affectivity, emotional predisposition, and attention, as well as to their preference for cognition. Overall, the EDICOP is, therefore, both relevant and useful, and further research on the mood–creativity link is merited to generate new contexts in higher education for the promotion of both the emotional and creativity dispositions and self-awareness, by combining three basic psychological processes (emotion, cognition, and motivation).

## 1. Introduction

The mood–creativity relationship is becoming increasingly recognized in a wide range of domains, especially due to its link to innovation in higher education students. In fact, the key to improving the efficiency of students and employees in education and work could rely on cultivating their emotional awareness, optimism, creative self-efficacy, critical thinking, social creativity, and emotional creativity [1,2,3]. Following Ivcevic and colleagues [4], emotions involve an experiential component (described by valence, activation, and regulatory focus), as well as abilities to understand, use, and manage these experiences in the service of thinking and creative work while creativity is defined as a process from the decision to be creative to idea generation and evaluation to product completion. Emotions are central to the creative process, from the emotion-filled decision whether to be creative, to positive emotions broadening thinking, and to inevitable frustrations on the way to creative achievement [4,5,6].On the one hand, studies included in the latest edition of the *Handbook of Vocational Education and Training* [7] consider creativity training to be the basis for integrating young people into the working world. In fact, creative individuals are understood to be the axis of new cultures of creativity and innovation, due to their personality traits, which include dynamism, proactivity, self-starting, openness, preference for novelty and challenges, prosocial orientation, team working, and sensitivity [8,9]. In this sense, Barabasch and Cattaneo [10] conclude that creative workers who can contribute to innovations are increasingly in demand, and both schools and training institutions need to respond to this shift in competence requirements by adjusting their pedagogical practices, including assessments, curricula, and learning environments. In fact, the European Community has launched many initiatives related to creativity, such as Creative Europe (2014–2020), and has directed efforts toward integrating the creativity of young people in the working context. Other researchers have included innovative methods in their higher education training programs, such as the applied neurocreativity program [11,12] or the personalized creativity learning system [13]. Moreover, new ways of thinking are included, like design thinking [14], game thinking and innovative thinking [15], and strategic thinking [16].

On the other hand, the results obtained in a multilevel meta-analysis suggest that emotional intelligence training should be considered an effective intervention in young adults [17]. In fact, emotional education facilitates the prevention of risk factors in young adults by improving their interpersonal relationships, mental health, and overall subjective well-being [18,19]; improving their academic outcomes and motivation to study [20]; and helping them to acquire the emotional abilities necessary to identify and manage the influence of emotions in information processing [21]. Following Keramanti and Gutkin [22], the homeostasis of the emotional stimuli can drive humans to create something, by motivating them to follow the shortest path in the space of physiological variables toward the desired setpoint. In other words, in creativity processes, seeking rewards could be equivalent to the fundamental objective of psychological stability. Taking into account the relevance of promoting motivational and socio-emotional abilities in higher education, some authors have proposed a list of content to include in all emotional intelligence programs [23,24]. Specifically, five core competencies proposed by The Collaborative for Academic, Social, and Emotional Learning (CASEL) have been applied to higher education populations: self-awareness, self-management, social awareness, relationship skills, and responsible decision-making. In contrast, poor emotional awareness can lead to symptoms of emotional distress, such as depression, anxiety, and stress, which are the most prevalent adjustment problems facing higher education students, and can have detrimental effects on academic functioning [25].

Despite the relevance of creativity training and emotional education among higher education students, we have found no intervention programs that combine both aspects in the same initiative. Thus, the main goal of this study is to design, implement, and evaluate experimentally the effects on higher education students of the Emotional Divergent–Convergent Thinking Program (EDICOP), which aims to promote both the emotional and creativity dispositions as well as self-awareness. 

### The Emotional Divergent–Convergent Thinking Program (EDICOP)

This intervention program includes assumptions from mood–creativity research [26,27] and theories related to creative thinking, emotion, and motivation [28,29,30]. The following models served as theoretical precedents for EDICOP: (1) “The creative emotions model” [31], (2) “The unifying model of the cognitive and affective-personal aspects of creativity” [32], (3) “The awakening creativity model” [33], (4) “The affect and creativity cycle model” [34], and (5) “The dual pathway to creativity model” [35]. Almost all of these study creativity as a dynamic process, taking the influence of emotions on the individual’s creative-thinking abilities into consideration.

The EDICOP intervention program consists of 16 activities divided into two areas (personal and professional) and five modules: (1) emotional damage to creativity, (2) emotional facilitation of creativity, (3) creating ideas, (4) evaluating and selecting ideas, and (5) presenting and defending ideas. All activities included in the intervention program and their specific goals are presented in Appendix A. 

The final goal of EDICOP training is to promote the emotional divergent and convergent thinking of the participants, specifically, by improving their emotional knowledge of creativity in the personal area (first part) and by applying affective-creative styles to the professional area (second part). The techniques used to develop emotional self-awareness in the first part (personal area) were reflexive role-taking, class debates, and changes in self-concept and self-image. The procedures selected to develop creative thinking in the second part (professional area) were (1) identification of each idea’s strengths and weaknesses; (2) making analogies by comparing different ideas and examining their similarities (e.g., metaphors, use of elements from nature); (3) imagination, lighting, and visualization; (4) checklisting, ideation, and brainstorming (e.g., de Bono’s five Thinking Hats); and (5) expressive actions like “ideart” (representing ideas in a work of art), storyboarding (writing a short script with the help of images), and moodboards (creating a collage with emotional expressions). 

## 2. Method

### 2.1. Evaluation Design

A quasi-experimental design with pretest–posttest measures and a non-equivalent control group was used. The criterion variables were the affective-creative styles (convergent-preventive style and divergent-proactive style), emotional creativity (emotional preparedness, novelty, and effectiveness/authenticity), emotional intelligence (emotional attention, clarity, and repair), positive affective disposition, and preference for cognition.

### 2.2. Participants

The sample consisted of 196 subjects (40% women and 60% men) aged between 16 and 24 years (Mage = 19; SD = 4.68). The participants were students at two vocational training centers in the Autonomous Community of the Basque Country (northern Spain). The sampling design employed was a non-probability convenience sampling, but we attempted to balance gender, age, and academic domain. In terms of the students’ academic domain, 38% had chosen to specialize in education sciences, 44% in scientific-technical sciences, and 18% in health sciences. Eighty percent of the participants had no work experience, while the rest gave private lessons to children, worked as servers in restaurants, or were dedicated to sports training. Regarding socioeconomic level, most participants (53.8%) had a medium-high level, 39.2% had a medium-low level, and 7% were from a low socioeconomic level. The participants were divided into two groups: 113 students were assigned to the experimental condition (63 females and 50 males; Mage = 19.35; SDage = 2.17), and 83 students were assigned to the control condition (48 females and 35 males; Mage = 20.03; SDage = 2.73). 

### 2.3. Instruments

To evaluate the program’s effect on the variables studied, the following instruments were administered before and after the program: the Emotion/Motivation-Related Divergent and Convergent Thinking Styles Scale (EDICOS) [2], the shortened Spanish version of the Emotional Creativity Inventory (ECI-S) [36], the Spanish version of the Trait Meta-Mood Scale (TMMS-24) [37], the Spanish version of the Positive and Negative Affect Scale PANAS [38], and the reduced Spanish version of the Need for Cognition Scale (NC) [39].

### 2.4. Emotion/Motivation-Related Divergent and Convergent Thinking Styles Scale (EDICOS)

The EDICOS [2] is a 30-item self-report questionnaire that provides information about consistent individual differences in emotional and motivational reactions to divergent and convergent thinking. The EDICOS includes four factors: convergent-preventive, divergent-proactive, convergent-unpleasant, and divergent-pleasant. The first two factors were selected for the present study: convergent-preventive style (e.g., “Considero interesante reflexionar sobre los problemas”/“I consider it interesting to reflect on problems”), and divergent-proactive style (e.g., “Me interesa participar en retos originales”/“I am interested in participating in original challenges”). Items are answered using a 6-point Likert scale, with options ranging from 1 (strongly disagree) to 6 (strongly agree). Both dimensions showed adequate psychometric properties, with Cronbach’s alpha values of α = 0.86 and 0.84, respectively.

### 2.5. Shortened Spanish Version of the Emotional Creativity Inventory (ECI-S)

The ECI-S [36] is a 17-item self-report questionnaire that provides information about emotional preparedness (e.g., “Pienso en mis reacciones emocionales e intento comprenderlas”/“I think about my emotional reactions and try to understand them”), emotional novelty (e.g., “He sentido emociones que probablemente otras personas no hayan experimentado jamás”/“I have felt emotions that other people have probably never experienced”), and emotional effectiveness/authenticity (e.g., “La forma en que expreso y experimento mis emociones me ayuda en mis relaciones con los/as demás”/“The way I express and experience my emotions helps me in my relationships with others”). Responses are given using a 6-point Likert scale, with options ranging from 1 (strongly disagree) to 6 (strongly agree). The Cronbach’s alpha values were appropriate for all dimensions of the ECI-S: preparedness (0.79), novelty (0.78), and effectiveness/authenticity (0.83).

### 2.6. Spanish Version of the Trait Meta-Mood Scale (TMMS-24)

Using three subscales, the TMMS-24 [37] is a self-report tool that assesses the extent to which people pay attention to and value their feelings (attention: e.g., “Pienso en mi estado de ánimo constantemente”/“I think about my mood constantly”), feel clear rather than confused about their feelings (clarity: e.g., “Casi siempre sé cómo me siento”/“I almost always know how I feel”), and use positive thinking to repair negative moods (repair: e.g., “Aunque a veces me siento triste, suelo tener una visión optimista”/“Although I am sometimes sad, I generally have an optimistic outlook”). It has 24 items to be answered on a 5-point Likert scale, with options ranging from 1 (strongly disagree) to 5 (strongly agree). The Spanish version of the TMMS showed adequate psychometric properties with Cronbach’s alpha values equal to or higher than *α* = 0.82 (0.82, 0.85, and 0.84, respectively). 

### 2.7. Spanish Version of the Positive and Negative Affect Scale (PANAS)

The Spanish PANAS [38] is a self-perception questionnaire with two main factors: positive affect and negative affect. Positive affect (PA) is a dimension of enthusiasm, activation, and alertness. High PA scores reflect a state of high energy, full concentration, and pleasurable engagement, whereas low PA is characterized by sadness and lethargy. Negative affect (NA) is a general dimension of subjective distress and unpleasant engagement. High NA scores include a variety of aversive mood states (such as anger, contempt, disgust, fear, and nervousness), while low NA reflects a state of calmness and serenity. The Spanish PANAS consists of two 10-item scales with options ranging from 1 (very little or nothing) to 5 (extremely). The instrument showed adequate internal consistency in its original version (PA, *α* = 0.85; NA, *α* = 0.81) and in the present research (PA, *α* = 0.76; NA, *α* = 0.82).

### 2.8. Reduced Spanish Version of the Need for Cognition Scale (NC)

The reduced Spanish NC [39] measures the extent to which individuals are motivated to think. Confirmatory factor analysis supports a two-factor structure composed of a positive factor related to the tendency to make mental effort (preference for cognition: e.g., “Me atraen más los problemas muy complejos que los sencillos”/“I am more attracted by highly complex problems than by simple ones”) and a negative factor related to the tendency to avoid and reject situations requiring sustained mental effort (avoidance of cognition: e.g., “Prefiero pensar el mínimo necesario en cada caso”/“I prefer to think the minimum necessary in each case”). The NC has 18 items (9 items per subscale), with options ranging from 1 (totally disagree) to 5 (totally agree). Thus, individuals are classified along a continuum ranging from high scores (individuals who enjoy performing cognitive activities) to low scores (individuals who avoid thinking except when it is required by situational demands). The NC showed adequate psychometric properties in the original version, with Cronbach’s alpha values of 0.83 (preference for cognition) and 0.74 (avoidance of cognition). In the present study as well, the preference and avoidance dimensions showed adequate internal consistency, with Cronbach’s alpha values of 0.84 and 0.76, respectively.

## 3. Procedure

A letter explaining the research project was sent to higher education centers. The directors of the centers were contacted, and interviews were held with those who agreed to participate. During the interview, the characteristics of the program were explained, and the directors were provided with the center’s authorization form and informed consent forms for participants in the experimental and control groups. Members of the research team administered a battery of assessment instruments to both groups as a pretest. 

The intervention program was implemented in the experimental group. The program consisted of 16 activities (Appendix A) divided into five modules designed to promote individuals’ affective-creative dispositions, emotional creativity, and emotional intelligence. The intervention was carried out by two psychologists previously trained in emotional education and creativity skills. While the experimental group received the designed program, the subjects in the control group continued with the activities already established within their regular academic curriculum. 

At the end of the program, as a posttest, the same battery of assessment instruments used in the pretest phase was administered to both the experimental and control groups in order to measure changes in the study’s variables. Additionally, a qualitative questionnaire was added in the posttest phase to examine subjective perceptions of possible changes in both samples (experimental and control). 

In accordance with the Declaration of Helsinki and following the ethical guidelines of the Ethics Committee for Human Research of the University of the Basque Country (UPV/EHU), we selected only students who wanted to participate in this study voluntarily. Informed consent was collected from the students themselves and the corresponding school authorities. 

## 4. Data Analysis

Data were analyzed using the IBM SPSS software (version 22). With the aim of examining the impact of the program on the variables studied, comparisons between the first (pretest) scores and the later (posttest) scores obtained after the implementation of the program were performed in the experimental group, using the Student’s *t-*statistic for related samples. The effect sizes associated with each comparison of means were calculated using the Hedges’ *g* statistic. The same analyses were performed when comparing the results yielded by the experimental and control groups, as well as when analyzing the effects of the program on males and females. 

## 5. Results

The mean scores and standard deviations yielded by the participants in the experimental group are shown in Table 1, specifically, those obtained before and after application of the program for the following variables: affective-creative styles (convergent-preventive style and divergent-proactive style), emotional creativity (emotional preparedness, novelty, and effectiveness/authenticity), emotional intelligence (attention, clarity, and repair), positive and negative affectivity, and preference for or avoidance of cognition. Additionally, the table shows the values obtained on the Student’s *t*-statistic and Hedges’ *g* statistic for comparison between the pretest and the posttest. 

As can be seen in Table 1, the EDICOP improved the students’ scores primarily in their preference for cognition (NC), positive affectivity (PANAS), and emotional preparedness (ECI-S), with effect sizes of notable magnitude (Hedges’ *g* = 0.53, 0.67, and 0.37, respectively). Moreover, values in the divergent-proactive dimension measured by the EDICOS also improved significantly after the program’s implementation, together with other ECI-S variables. Finally, it should be noted that the program also had a certain effect on emotional attention as measured by the TMMS-24, with a moderate effect size even though the results did not reach statistical significance. 

Table 2 presents the mean scores and standard deviations yielded by the experimental and control group participants in all variables measured before (pretest phase) and after (posttest phase) the program’s implementation, as well as the *F* values obtained for the Analysis of Variance (ANOVA) and Hedges’ *g* statistic for the comparison between the two groups.

ANOVA revealed that the experimental group’s scores were significantly higher than those of the control group, especially in emotional preparedness (ECI-S), followed by preference for cognition (NC), with notable magnitudes of both differences (Hedges’ *g* = 0.48 and 0.46, respectively). Moreover, the experimental group showed differences that were statistically significant and of a moderate magnitude with respect to the control group in the divergent-proactive dimension measured by the EDICOS. The program had a lower impact on positive affect as measured by the PANAS, which had a big effect size (Hedges’ *g* = 0.65), and also on emotional attention (TMMS-24) and novelty (ECI-S), with moderate effect sizes.

## 6. Effects of the Program on Males and Females

To explore possible sex differences in the EDICOP, we carried out a pretest Multivariate Analysis of Variance (MANOVA) with the set of variables assessed, the results of which, *F*(1,53) = 1.50, *p* > 0.05, showed that before the intervention, both sexes had similar levels of creative-affective styles, positive–negative affectivity, emotional creativity, emotional intelligence, and disposition toward cognition. To assess whether the program had a different effect based on sex, that is, to analyze whether the program stimulated a higher level of change in males or in females, or whether both sexes were affected similarly, we carried out a pretest–posttest MANOVA, the results of which (*p* > 0.05) were non-significant, with a medium effect size in all variables, and pointed in the same direction (*p* > 0.05). Therefore, these results indicate that the changes stimulated by the intervention were similar in both sexes. In fact, there were no statistically significant differences between the experimental males and females on any of the indicators, nor were any statistically significant differences found in the changes they experienced as an effect of the program. Therefore, the intervention did not have an impact that was differential as a function of sex.

## 7. Discussion and Conclusions

Individuals’ emotional skills and self-knowledge are considered to be key elements for well-being, learning processes, social relationships, and prevention of risk factors. Creativity abilities are considered relevant for students of employment age to adapt to the ever-changing work context. However, many students in higher education tend to pay more attention to their social and academic self-concept than to their emotional self-concept, and they tend to need additional information related to the influence of emotions on the cognitive process (including creative thinking). Nevertheless, there are few educational programs that include training in creativity skills together with personal-affective factors. Thus, in the present study, we designed, implemented, and evaluated the Emotional Divergent–Convergent Thinking Program (EDICOP). 

The EDICOP aims to incorporate two central concepts found in the literature to be relevant in higher education [31,32,33,34,35]: emotional education and creativity training. Specifically, the final goal of the 16 activities included in the program was the promotion of both the emotional and creativity dispositions and self-awareness.

From the results obtained in this study, it can be concluded that the program’s application improved the students’ positive affectivity, divergent-proactive style, preference for cognition, and emotional preparedness. These results are consistent with those of other studies that have found an association between positive emotions and divergent thinking [28,35]. The tendency to feel positively and to be in a positive mood might reflect the optimal mood state for divergent thinking (i.e., tasks requiring flexibility and intuition). In addition, individuals with high scores in preference for cognition tend to have fun performing cognitive activities [40]. Ritter and Ferguson [41] explain that a positive attitude facilitates divergent thinking, compared to a neutral control condition. Otherwise, participants in the present study did not improve their scores in either negative affectivity or convergent-preventive style. This causes us to think that the design of the EDICOP has special sensitivity with regard to the promotion of positive affectivity, divergent thinking, and proactive style (Modules II and III) in comparison to the training of negative affectivity, convergent thinking, and preventive style (Modules I and IV). In fact, there was a broad margin of improvement in Module V with respect to the phase that combines divergent and convergent thinking processes, as the program requires the participants to carry out a final activity that combines originality and effectiveness. 

In addition, the program had a certain influence on the students’ emotional intelligence, especially with regard to the emotional attention dimension. This result is in line with the approach based on the emotional intelligence model presented by Goleman and colleagues [42], in which self-awareness is one of the main emotional competences, and it may provide new evidence about the relationship between creativity and the ability to perceive feelings of oneself [43,44,45].

Based on affective information processing theory, emotional intelligence allows an individual to maintain a more positive affect when facing a complex problem situation, while emotion attention (emotional intelligence) and emotion preparedness (emotional creativity) enable one to utilize a positive affect to promote creativity [46]. The above findings confirm the positive effect of optimism, emotional attention, and preparedness on creativity, and more specifically on divergent thinking and preference for cognition [47]. From the perspective of a brain structural basis, creative thinking processes are associated with emotion-related brain structures, such as the regional grey matter volume of the hippocampus and amygdala [48]. Taken together, the above findings may reveal a close relationship between creativity and affectivity, and the underlying similar brain structures involved in both. 

It is necessary to reach a more profound comprehension about the mood–creativity relationship in teenagers and young adults, which should involve more interdisciplinary research, based on a systematic view of emotional education together with emotional creativity that acknowledges a series of interrelated strengths that could operate at different levels. Within this multidisciplinary research context, the following future lines of research are proposed: (1) the implementation and assessment of the EDICOP in other domains, such as the artistic and dramatic sciences, (2) the adaptation of the contents of the EDICOP to other age groups, such as students in secondary education, (3) a comparison of the effects of EDICOP cooperative activities by adapting the program to include competitive activities, (4) the exploration of the maintenance of long-term effects of the program (longitudinal data collection), (5) the effect of contextual factors as mediator variables such as the group emotional climate, and (6) the evaluation of specific techniques used along with the intervention program: brainstorming, de Bono’s Thinking Hats, the SCAMPER technique (Substitute, Combine, Adapt, Modify, Put to another use, Eliminate, and Reverse), Osborn creative problem-solving process (Fact finding, Idea finding, and Solution finding), SWOT analysis (Strength, Weakness, Opportunity, and Threat), and storyboarding. 

Moreover, to prevent possible mono-method bias caused by the use of self-report instruments in this study, it may be useful to include performance tests in the evaluation system of the EDICOP, such as the Torrance Test of Creative Thinking (TTCT-Figural Form B) [49,50] —which examines fluency, originality, and elaboration—and the Emotional Consequences Test (ECT) [51]—which examines the fluency, flexibility, and originality of responses given in emotional situations and the emotions (pleasant or unpleasant) felt in each situation. Furthermore, there are many available methods that are designed to improve quasi-experiments (e.g., propensity scores) that should be considered to strengthen the findings here. Especially given the focus on self-reporting, it is important to control for measurement error as much as possible, for example, through the use of structural equation modeling (SEM) analysis. 

The present results indicate that the design, implementation, and assessment of the EDICOP is useful for both educational practice and research. Educators may find the study data useful for designing other training materials and intervention programs in the educational context to improve emotional awareness applied to creativity processes. Finally, these results may help to advance our understanding of the dynamic interplay of cognitive, emotional, and motivational factors in developing and improving human creativity. 

## Figures and Tables

**Table 1:** Mean scores and standard deviations and Student’s *t*-statistic, *F, p-values,* and Hedges’ *g* statistic values yielded during the pretest and posttest phases by the experimental group (*n* = 113).

	Pretest		Posttest		Analysis of Variance (ANOVA) Pre–Post			
	*M*	*SD*	*M*	*SD*	*F*	*p*	*t*	*g*
Convergent-preventive style	33.8	7.87	34.2	5.7	1.12	0.83	0.21	0.05
Divergent-proactive style	23.05	4.61	24.93	4.93	0.07	0.02	1.22	0.39
Emotional preparedness	48.72	10.34	52.8	11.68	5.15	0.01	2.61	0.37
Emotional novelty	55.78	14.37	59.45	12.39	0.83	0.05	1.08	0.27
Emotional effectiveness/authenticity	40.55	12.39	44.21	14.37	0.85	0.05	1.07	0.27
Emotional attention	26.45	6.21	28.86	5.77	0.182	0.07	0.28	0.39
Emotional clarity	28.05	3.88	28.74	6.33	1.75	0.63	0.47	0.17
Emotional repair	26.65	5.21	28.17	5.18	0.021	0.24	1.18	0.29
Positive affect	26.34	5.1	29.68	5.67	0.172	0.01	1.28	0.62
Negative affect	26.05	3.88	25.47	5.33	0.175	0.21	1.47	0.12
Preference for cognition	13.10	2.31	14.54	3.11	4.31	0.001	3.31	0.53
Avoidance of cognition	15.24	3.31	15.9	3.09	0.08	0.43	2.05	0.20

**Table 2:** Mean scores and standard deviations, and Student’s *t*-statistic, *F, p-values*, and Hedges’ *g* statistic values obtained by the experimental and control group participants during the pretest and posttest phases.

	Experimental (*n* = 113)				Control (*n* = 83)				Experimental—Control (*n* = 196)							
	Pretest		Posttest		Pretest		Posttest		ANOVA Pretest				ANOVA Posttest			
	*M*	*SD*	*M*	*SD*	*M*	*SD*	*M*	*SD*	*F*	*p*	*t*	*G*	*F*	*p*	*t*	*g*
Convergent-preventive style	33.8	7.87	34.2	5.7	33.69	7.29	33.87	7.49	2.11	0.40	0.11	0.03	1.12	0.43	0.13	0.05
Divergent-proactive style	23.05	4.61	24.93	4.93	22.9	4.47	23	4.68	0.70	0.22	0.21	0.19	0.07	0.02	1.22	0.39
Emotional preparedness	48.72	10.34	52.8	11.68	46.08	9.98	47.42	10.32	3.23	0.41	0.58	0.18	4.23	0.001	2.54	0.48
Emotional novelty	55.78	14.37	59.45	12.39	55	13.01	55.43	13.05	0.63	0.59	0.32	0.13	0.73	0.049	1.12	0.31
Emotional effectiveness/authenticity	40.55	12.39	44.21	14.37	41.05	11.93	42.56	14.06	0.54	0.77	0.19	0.1	0.74	0.074	1.16	0.11
Emotional attention	26.45	6.21	28.86	5.77	26	6.01	27.22	4.82	0.19	0.34	0.14	0.03	0.28	0.04	0.24	0.30
Emotional clarity	28.05	3.88	28.74	6.33	27.9	3	28.01	5.94	0.78	0.63	0.27	0.11	1.75	0.36	0.47	0.11
Emotional repair	26.65	5.21	28.17	5.18	25.91	4.86	27.88	5.20	0.01	0.44	0.21	0.05	0.021	0.64	1.12	0.05
Positive affect	26.34	5.1	29.68	5.67	26.1	4.9	26.04	5.49	0.18	0.34	0.28	0.15	0.189	0.04	1.28	0.65
Negative affect	26.05	3.88	25.47	5.33	27	3.1	26.95	4.88	0.11	0.67	0.31	0.08	0.181	0.27	1.47	0.28
Preference for cognition	13.10	2.31	14.54	3.11	12.83	2.1	13.10	3.12	2.11	0.51	0.24	0.16	3.31	0.01	2.74	0.46
Avoidance of cognition	15.24	3.31	15.9	3.09	15.45	3.12	15.54	3.32	0.08	0.63	0.05	0.01	0.08	0.43	2.05	0.11

## Appendix A

**Table A1:** Structure, activities, and objectives of the EDICOP.

**PART I: Emotional Awareness around Creativity (Personal Area)**					
**What Damages Creativity? (Module I)**			**What Facilitates Creativity? (Module II)**		
**Name of the Activity**	**Goals of the Activity**		**Name of the Activity**	**Goals of the Activity**	
False beliefs	- Identify and externalize negative prejudices toward creativity. - Be aware of the damaging influence of such prejudices on creativity.		Brain test	- Assess the cognitive processes included in both brain hemispheres and understand the influence of each hemisphere on creativity.	
Face to face with creativity	- Define one’s own self-image (positive or negative) with regard to creativity and reinforce underestimated creative abilities.		Creative self-concept	- Identify the characteristics of the creative personality that fits with one’s self.	
The sound of embarrassment	- Identify the embarrassment that a group creativity task might cause.		Heart-storming	- Reflect on the effects of having an optimistic attitude during a divergent-thinking task.	
Pass the emotion	- Identify the negative influence of time pressure on concentration capacity.		Beats	- Identify the facilitating influence of a positive self-concept (self-esteem) on group creativity.	
**PART II: Emotions Applied to Creativity Training (Professional Area)**					
**When Creating Ideas (Module III)**		**When Evaluating and Selecting Ideas (Module IV)**		**When Presenting and Defending Ideas (Module V)**	
**Name of the Activity**	**Goals of the Activity**	**Name of the Activity**	**Goals of the Activity**	**Name of the Activity**	**Goals of the Activity**
Thinking hats	- Use pleasant emotions when creating new ideas. - Know the de Bono’s Thinking Hats technique and use it proactively.	Emo-exam	- Use unpleasant emotions when evaluating ideas. - Know the SCAMPER and OSBORN techniques, and use them with a motivation to prevent failures.	The scriptwriter	- Be able to visualize group achievements. - Know and use the storyboarding and moodboard techniques.
Bionics	- Use pleasant emotions when combining ideas. - Know the Bionic technique and use it proactively.	SWOT analysis	- Use unpleasant emotions when analyzing the strengths and weaknesses of an idea. - Use unpleasant emotions when making decisions about ideas. - Know the SWOT technique and use it with a motivation to prevent failures.	Ideart	- Design an emotionally creative logo of the final idea. - Know and use the Ideart technique.
Slogan with sense	- Use pleasant emotions when noticing an idea coming from different senses. - Know the Five Senses technique and use it proactively.			Emo-create!	- Be able to “sell” a business idea that represents emotions and creativity and combines originality and effectiveness.
